# Supplementary material for: A UMLS-based spell checker for natural language processing in vaccine safety
Source: BMC Med Inform Decis Mak. 2007 Feb 12;7:3. doi: 10.1186/1472-6947-7-3 (PMC1805499; doi:10.1186/1472-6947-7-3)
Supplement: Additional file 13 — RAP application directory. Sets up directory for RAP (RDF application for PHP) source code [file 1472-6947-7-3-S13.gz › rap/doc/phpdoc/li_default.html]

xml version="1.0" encoding="iso-8859-1"?


default

Description
:   Class trees  
    Index of elements  
    Todo List

Classes
:   ATOM
:   ATOM\_RES
:   DC
:   DC\_RES
:   FOAF
:   FOAF\_RES
:   OWL
:   OWL\_RES
:   RDF
:   RDFS
:   RDFS\_RES
:   RDF\_RES
:   RSS
:   RSS\_RES
:   VCARD
:   VCARD\_RES

Files
:   ATOM\_C.php
:   ATOM\_RES.php
:   constants.php
:   DBase.php
:   DC.php
:   DC\_C.php
:   DC\_RES.php
:   FOAF.php
:   FOAF\_C.php
:   FOAF\_RES.php
:   InfModelP.php
:   ModelP.php
:   OWL.php
:   OWL\_C.php
:   OWL\_RES.php
:   RDF.php
:   RdfAPI.php
:   RDFS.php
:   RDFS\_C.php
:   RDFS\_RES.php
:   RDF\_C.php
:   RDF\_RES.php
:   RDQL.php
:   ResModelP.php
:   RSS.php
:   RSS\_C.php
:   RSS\_RES.php
:   SyntaxGRDDL.php
:   SyntaxN3.php
:   SyntaxRDF.php
:   Utility.php
:   VCARD.php
:   VCARD\_C.php
:   VCARD\_RES.php
:   Vocabulary.php
:   VocabularyClass.php
:   VocabularyRes.php

phpDocumentor v 1.3.0RC3
